# Effectiveness of nudge-based visual storytelling in antibiotic adherence in uncomplicated urinary tract infection in Pakistan: protocol for a randomized controlled trial

**DOI:** 10.1186/s13063-025-09328-1

**Published:** 2025-12-17

**Authors:** Iltaf Hussain, Muhammad Fawad Rasool, Jamshid Ullah, Inzemam Khan, Muhtar Kadirhaz, Miaomiao Xu, Chengzhou Tang, Yi Dong, Wei Zhao, Faiz Ullah khan, Jie Chang, Yu Fang

**Affiliations:** 1 Department of Pharmacy Administration, School of Pharmacy, Xi’an Jiaotong University, Xi’an, China; 2 Center for Drug Safety and Policy Research, Xi’an Jiaotong University, Xi’an, China; 3 Shaanxi Center for Health Reform and Development Research, Xi’an, China; 4 Research Institute for Drug Safety and Monitoring, Institute of Pharmaceutical Science and Technology, Western China Science and Technology Innovation Harbor, Xi’an, China; 5 Department of Pharmacy Practice, Faculty of Pharmacy, Bahauddin Zakariya University, Multan, Pakistan; 6 Department of Medical Laboratory Technology, Institute of Paramedical Sciences, Khyber Medical University, Peshawar, Pakistan; 7 Department of Pharmacy, University of Peshawar, Peshawar, Pakistan; 8 Department of Pharmacy Administration and Clinical Pharmacy, School of Pharmaceutical Sciences, Peking University Health Science Center, Peking University, Beijing, China; 9 International Research Center for Medicinal Administration (IRCMA), Peking University, Beijing, China

**Keywords:** Nudge, Visual storytelling, Antibiotic adherence, Urinary tract infection

## Abstract

**Background:**

Antibiotic resistance (ABR) is a global health threat, significantly driven by its misuse. The World Health Organization stresses the need for better antibiotic use to combat ABR through behavioral change interventions. Visual storytelling, which merges narrative with visuals, can enhance health behaviors by boosting cognitive and emotional engagement. In Pakistan, more than half of the population lack access to mobile phone, limiting digital health solutions. To address this gap, we developed a culturally tailored Visual Storytelling for Antibiotic Adherence (VISTA) intervention – a theory-driven sticker-based tool – to improve antibiotic adherence among urinary tract infection (UTI) patients in low-connectivity settings.

**Methods:**

In this parallel, two-arm superiority randomized controlled trial, participants with physician-confirmed uncomplicated UTI who have filled an oral antibiotic prescription will be recruited from six tertiary care hospitals in Khyber Pakhtunkhwa, Pakistan. The participants will be randomized in a 1:1 manner to receive either the VISTA intervention or standard care. The VISTA intervention was developed based on nudge theory, guided by the Taxonomy of Choice Architecture framework, and complemented by the MINDSPACE (messenger, incentives, norms, defaults, salience, priming, affect, commitments, and ego) framework. It was developed and validated through a Delphi method, assessing expert feedback, and refined based on a patient understanding study. The intervention sticker uses contrasting colors—red (non-adherence: bacteria evolving into “superbugs”) and green (adherence: antibiotics fully eradicating bacteria)—to communicate the consequences of incomplete versus complete adherence. The primary outcome is adherence, measured by pill count at the initial follow-up. Secondary outcomes include UTI recurrence, knowledge, and attitudes regarding antibiotic adherence. Analysis will follow both intention-to-treat and per-protocol principles.

**Discussion:**

This randomized controlled trial will evaluate a theory-driven, scalable intervention for low-resource, low-connectivity settings, aligning with WHO priorities for ABR mitigation. It addresses the patient support gap caused by limited digital access. Pill counts provide an objective adherence measure, though they may miss intentional non-adherence; including UTI recurrence as a secondary outcome helps mitigate this. Cultural tailoring through expert panels and patient feedback aims to enhance relevance and applicability across similar contexts, improving generalizability.

**Trial registration:**

The trial was registered with ClinicalTrials.gov (registered: March 13, 2025, NCT06885658, https://clinicaltrials.gov/study/NCT06885658).

**Supplementary Information:**

The online version contains supplementary material available at 10.1186/s13063-025-09328-1.

## Introduction

### Background

Antibiotic resistance (ABR) presents significant global public health threats, and it is estimated that antibiotic-resistant infections will lead to 10 million deaths each year by 2050 [1]. The overuse and misuse of antibiotics play a significant role in the development of ABR [2–4]. Antibiotic misuse from the demand side includes practices such as obtaining antibiotics without a prescription, self-medication, and failing to adhere to the prescribed treatment, which involves skipping doses or not completing the antibiotic course [5, 6]. To combat the impending threat of ABR, the World Health Organization (WHO) has identified improving rational antibiotic use among individuals as the most essential policy target to mitigate ABR [7].

Non-adherence to antibiotic therapy, a pervasive yet comparatively under-prioritized form of misuse, constitutes a major ABR driver. This behavior stems from multifaceted barriers including forgetfulness, regimen misunderstanding, insufficient awareness about the consequences of incomplete treatment [6, 8], premature discontinuation upon symptom relief, or dose omission due to side effects or financial constraints [6, 9], and comprehension challenges among individuals with limited health literacy, particularly in resource-poor settings [9, 10]. Studies confirm that non-adherence accelerates resistance evolution, as incomplete treatment courses enable bacterial survival and select for resistant strains, progressively eroding antibiotics’ efficacy [11, 12]. Thus, enhancing patient adherence to prescribed antibiotic regimens aligns with WHO’s individual-focused mitigation strategy and is essential to preserve therapeutic effectiveness.

To address these behavioral challenges, our intervention leverages “nudge” theory advocated by Thaler and Stain in 2008, which is a framework proposing that subtle choice architecture modifications can predictably influence decisions while preserving autonomy [13]. While prior nudges primarily targeted prescriber behavior, for instance, social norm feedback reducing antibiotic prescriptions [14], we apply this approach to patient adherence. Specifically, we employ visual storytelling—a nudge-aligned technique using narrative and visual cues to guide antibiotic adherence decisions. This approach has proven effective in fostering adherence behavior by engaging both cognitive and emotional pathways, thus enhancing the patient’s understanding of antibiotics and encouraging actions that mitigate resistance [15–17]. Moreover, visual storytelling can help to overcome socioeconomic barriers in low-resource settings by offering a cost-effective way to educate patients about the risks of ABR and the importance of completing prescribed antibiotic courses. This ultimately contributes to improved health outcomes and reduced resistance rates [18].

### Rationale and research question

Visual storytelling enhances antibiotic adherence by cognitively and emotionally engaging patients, transforming complex medical information into more accessible and memorable visual narratives. By combining narrative elements with visual cues, visual storytelling helps patients understand causal relationships between non-adherence and consequences, including ABR and treatment failure, and highlights the importance of completing their prescribed antibiotic courses, which is crucial in preventing ABR [17]. Additionally, emotional involvement fosters empathy or concern, motivating patients to adhere more closely to their treatment regimens [19].

In low-resource settings, where digital strategies, such as Short Message Service (SMS) and smartphone applications, face limited feasibility given disparate mobile phone and smartphone access, visual storytelling provides an important solution that is scalable and affordable [20, 21]. Notably, while mobile phone ownership is 78% in LMIC [22], in Pakistan, only 46% of the population owns a mobile phone, and this rate is even lower in rural areas and for smart phone ownership [23]. By emphasizing non-digital methods, the intervention ensures inclusiveness for over half the population without mobile phone access, addressing systemic healthcare inequalities.

Visual storytelling’s ability to simplify and visualize complex medical information and arise emotional responses holds potential for fostering long-term behavioral changes, particularly in populations with limited access to digital tools. This approach aligns with the need to address antibiotic adherence disparities in resource-constrained settings. While storytelling has been effectively applied in domains like vaccination promotion [8], improving psychological outcomes [9–12], and management of anxiety in cardiac ICU [13] and dental pain [24], its potential to improve antibiotic adherence remains unexplored.

As such, this current study will employ a randomized controlled trial (RCT) design to evaluate whether a sticker-based Visual Storytelling for Antibiotic Adherence (VISTA) intervention improves antibiotic adherence compared to standard care among adults with uncomplicated urinary tract infections (UTIs). Our research question's feasibility, significance, novelty, ethical considerations, and relevance are framed according to the FINER criteria.

## Method

The protocol was reported in accordance with Standard Protocol Items: Recommendation for Interventional Trials (SPIRIT) [25].

### Study design

**Fig. 1:**
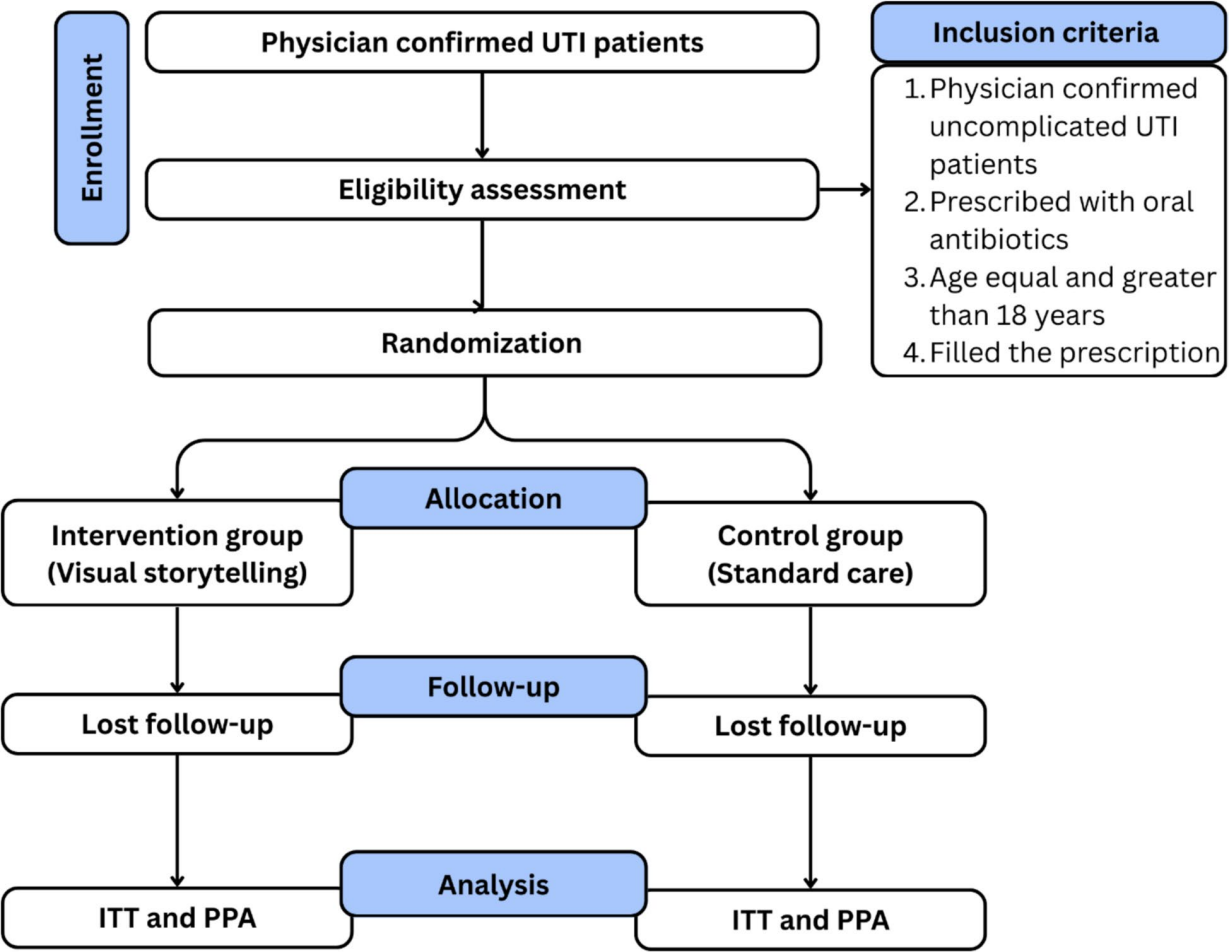
Schematic flow diagram of the trial. ITT intention-to-treat analysis, PPA per-protocol analysis

The study will employ a parallel two-arm superiority RCT design to test whether the VISTA intervention results in higher antibiotic adherence than standard care. Participants will be randomly allocated in a 1:1 ratio to either the intervention group or the control group. For the control group, participants will receive routine care, typically verbal or written instructions about antibiotic use, because it reflects the real-world standard practice for UTI treatment in Pakistan, whereas the intervention group will include VISTA stickers on the antibiotic boxes to improve antibiotic adherence. Both groups will be monitored for their adherence to the prescribed antibiotic course. The details can be seen in Fig. 1.

### Study setting

**Fig. 2:**
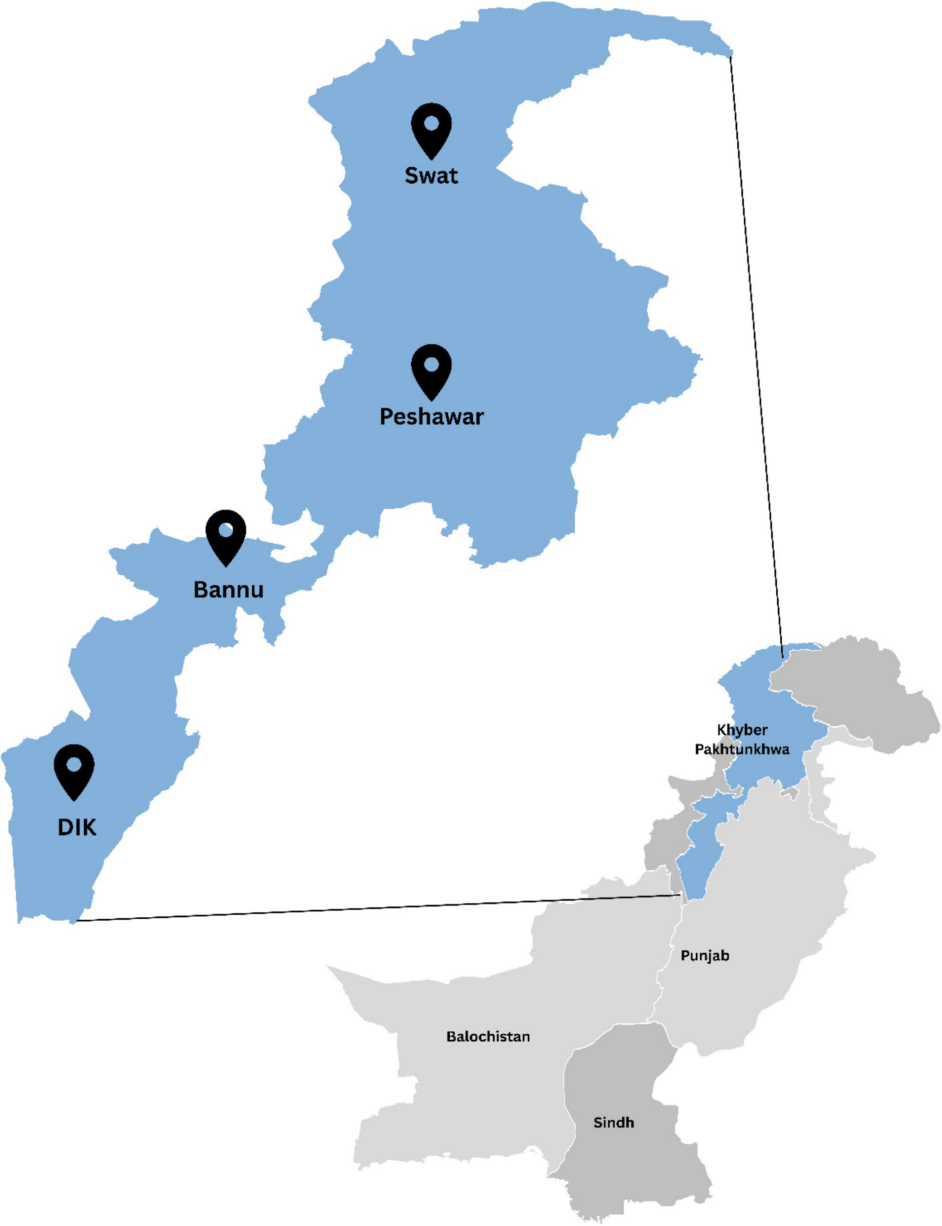
Study area for the current study. DIK Dera Ismail Khan

The study setting will be tertiary care hospitals in the Khyber Pakhtunkhwa province of Pakistan. According to the health department of Khyber Pakhtunkhwa, there are nine tertiary care hospitals (healthkp.gov.pk/#). For this study, six hospitals were selected based on their willingness to participate and support the research (Peshawar, *n* = 3,; Swat, *n* = 1,; Bannu, *n* = 1,; Dera Ismail Khan, *n* = 1). Three hospitals in Peshawar represent the main urban centers of the province. Two hospitals in Bannu and Dera Ismail Khan cover the southern rural districts, while the Saidu Group of Teaching Hospitals, Swat, represents a semi-urban site serving surrounding rural communities in northern Khyber Pakhtunkhwa. Together, these sites provide a mix of urban and rural populations, reflecting the province’s geographic and socioeconomic diversity. Formal permissions were obtained from the respective hospital administrations to conduct the study at these sites. The selected study areas are given in Fig. 2. In each selected hospital, patients from the urology/nephrology outpatient department (OPD) will be selected based on physician diagnosis.

### Patients’ population and sample size

Participants will include adults (≥ 18 years) with physician-diagnosed uncomplicated UTI who meet all inclusion criteria: (1) confirmed UTI diagnosis, (2) prescribed oral antibiotics (tablet or capsule), (3) having obtained their prescribed medication, (4) willing to participate, and (5) able to provide informed consent.

Patients with polypharmacy (taking five or more medications) [26] are excluded to minimize the risk of confounding factors related to multiple treatment regimens and potential medication interactions that could affect adherence. Patients with complicated UTI (pyelonephritis, sepsis, structural abnormalities) and with multiple comorbidities will be excluded. In addition, patients with cognitive impairment and pregnant or breastfeeding women will also be excluded from the study. Participants may discontinue participation at any time upon request or if they experience clinical deterioration or become ineligible (hospitalization or transfer). All discontinuations and reasons will be documented and summarized in the CONSORT flow diagram.

Eligible uncomplicated UTI patients will be approached during their outpatient visits in the urology department, and written informed consent will be obtained before participation. The patients will be recruited in the current study using the SEAR (Screened, Eligible, Approached, Randomized) framework [27].

The sample size was calculated using R statistical software (version 4.3.0) with “pwr” package (version 1.3.0). Based on our pilot study findings, which demonstrated a 16% improvement in adherence (64.3% in the intervention group vs. 48.3% in the control group), we used this observed effect size (Cohen’s *h* = 0.32) for power calculation. To detect this clinically significant difference in adherence rates with 80% power at a 5% significance level (two-sided test), 152 participants per group (304 total) would be required. Accounting for an estimated 15% attrition rate, informed by our pilot study, we will enroll 179 participants per group (358 total).

### Intervention development

**Fig. 3:**
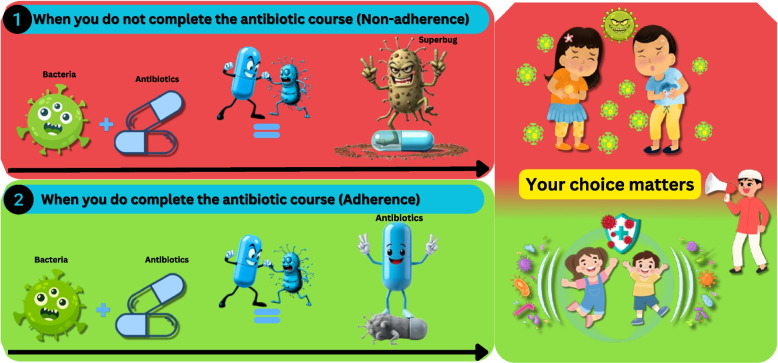
Nudge-based developed intervention sticker. This intervention sticker, designed for antibiotic packaging, visually contrasts non-adherence (up) and adherence (down) to emphasize the consequences of stopping treatment early. The upper panel illustrates how incomplete antibiotic use allows bacteria to evolve into “superbugs,” while the down panel reinforces that completing the full course eradicates infection and protects others. The accompanying illustration “Your choice matters” integrates cultural themes of family protection and social responsibility

The VISTA intervention was developed using a multi-framework behavioral design grounded in the concept of nudge theory (Fig. 3). Nudge theory, proposed by Thaler and Sunstein in 2008, provided the theoretical foundation and suggested that subtle changes in the way choices are presented or structured can influence the decisions and behavior of people predictably without restricting their freedom of choice [13]. The Taxonomy of Choice Architecture framework [28] was used to systematically categorize the choice architecture techniques, and translated this theory into a practical design strategy by categorizing nudge techniques, such as information simplification and visual reminders, through sticker placement. The messenger, incentives, norms, defaults, salience, priming, affect, commitments, and ego (MINDSPACE) framework complemented these structural techniques by identifying the underlying psychological drivers including credible messenger and affective appeal to enhance engagement and adherence motivation. Together, these frameworks ensured that the VISTA intervention was both theoretically grounded and behaviorally optimized for the context-specific decision-making environment [29].

The VISTA intervention sticker uses two contrasting scenarios to illustrate the consequences of non-adherence (upper red panel) versus adherence (lower green panel). The red panel depicts bacteria evolving into “superbugs” when treatment is stopped early, while the green panel shows antibiotics fully eradicating bacteria when the full course is completed. The patients in the intervention group will receive this intervention as a sticker on their antibiotic packs.

The intervention was developed in two steps.

#### Step 1: the intervention development using the Delphi method

The Delphi method was used to develop and refine the intervention through iterative rounds of expert feedback [30] and was conducted between November 2024 and February 2025. A total of 40 panelists participated in this step. The panelists were based on clinical expertise (≥ 5 years of experience in treating UTIs), research expertise (published work related to health communication, public health, patient adherence, or antimicrobial resistance), and geographic representation (50% of experts were from Pakistan to ensure feasibility and cultural relevance). Experts were identified through academic databases, professional networks, and snowball sampling.

**Fig. 4:**
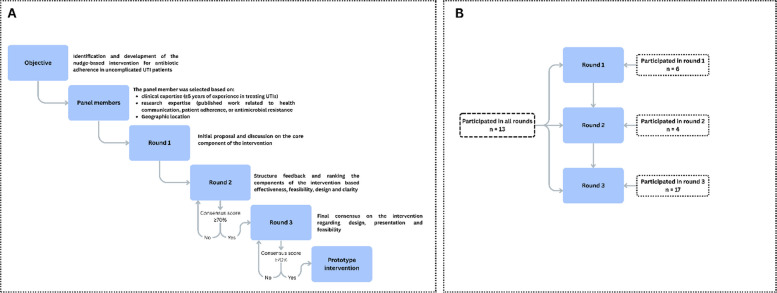
Delphi process for the intervention development (**A**) and panelist participated in the development process (**B**)

The process was completed in three rounds (round 1: initial proposal and discussion on the core component of the intervention; round 2: structure feedback and ranking the components of the intervention, based on effectiveness, feasibility, design, and clarity; round 3: final consensus on the intervention regarding design, presentation, and feasibility). An agreement was defined as ≥ 70% of experts rating a component as essential or acceptable [31]. Components that failed to meet the threshold after round 3 were discarded. The details can be seen in Fig. 4.

#### Step 2: intervention understanding study: patients’ perspective

In this step, the patients’ understanding of the prototype intervention, developed through the Delphi method of step 1, was assessed using a mixed method study design. The study was conducted between March and May 2025. The study participants were adult patients diagnosed with UTI. A total of 30 participants were selected from the outpatient department of tertiary care hospitals, Khyber Pakhtunkhwa, Pakistan.

**Fig. 5:**
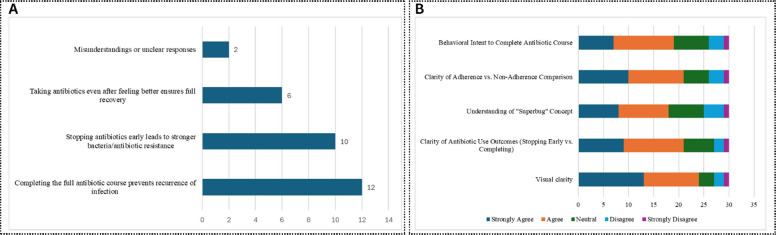
Patients’ understanding of the main message (**A**) and visual clarity (**B**) of the intervention

The quantitative analysis showed that 93.3% of the patients correctly identified the main message of the visual story, highlighting the overall clarity of the intervention (Fig. 5A). Regarding the overall clarity of the intervention visual, 80% of the participants agreed or strongly agreed that the visuals were clear, and 70% stated that there was a clear visual scenario between course completion and incompletion. The details can be seen in Fig. 5B. The demographic analysis showed that 40% of the participants were housewives, 56.7% resided in rural areas, and 20% had no formal education, underscoring that the intervention was a low-literacy-friendly design.

**Fig. 6:**
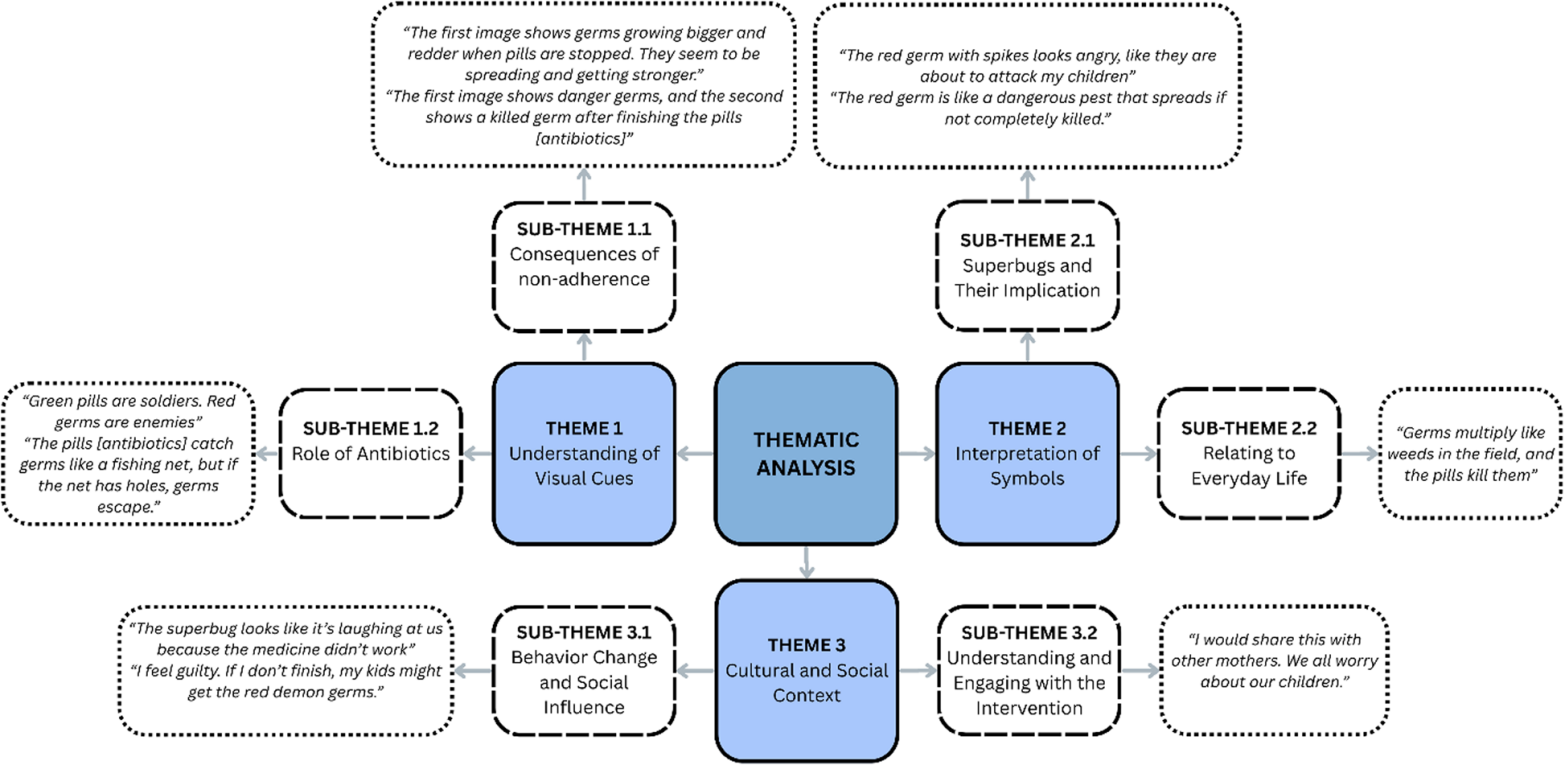
Thematic analysis of the patient interviews

The qualitative analysis identified three main themes: (1) understanding of the visual cues: participants linked non-adherence to stronger germs (e.g., “*Germs grow bigger and redder when pills [antibiotics] are stopped*”), (2) interpretation of symbol: “superbug” was metaphorically understood as weeds, or stubborn goats, (3) cultural and social context: metaphors resonated when tied to daily life (e.g., “*Germs multiply like weeds*”), and social responsibility emerged as a motivator (e.g., “*Finishing pills [antibiotics] protects my family*”). The details are shown in Fig. 6. These insights shaped the intervention’s final design, ensuring clarity, cultural relevance, and behavioral alignment, validating its readiness for the RCT.

### Randomization

The participants will be randomly assigned to either the VISTA intervention or control (standard care) groups within each hospital using block randomization. Based on the number of hospitals, a unique randomization list will be generated for each hospital. The block size will be six to ensure balanced allocation within each hospital. The block randomization will be conducted through R using the “blockrand” package. An independent researcher, who has no role in participant recruitment or intervention delivery, will generate the list.

### Allocation

The allocation sequence will be generated by R, and the list will be provided to the data collectors. To ensure allocation concealment, sequentially numbered, opaque, sealed envelopes (SNOSE) will be used, which will be opened only after a participant is deemed eligible and has consented. Personnel responsible for screening and enrollment will not have access to the randomization sequence prior to allocation; only the statistician generating the sequence will have access.

To avoid duplication, the “make answer unique” option will be activated in the Epicollect5 data collection tool. The data will be checked weekly to ensure balance between intervention and control groups, using Microsoft Excel. If there is a minor imbalance (55:45), data collection will continue as block randomization will correct itself over time. However, if there is a major imbalance (70:30), the data collection process audit will be carried out, and if necessary, the randomization list will be regenerated for that hospital.

### Blinding

The study participants and site staff delivering the intervention will remain unblinded due to the nature of the behavioral intervention. The individual blinded to the intervention assignment will carry out the data analysis using the neutrally labeled data set, such as “group A” and “group B.” To ensure consistent and objective measurement of the outcomes, site staff will receive training to rigorously follow the protocol, and regular monitoring and audits will be conducted to ensure compliance. The physician responsible for patient diagnosis and confirming UTI recurrence will remain blinded to the intervention assignment.

### Delivery

**Fig. 7:**
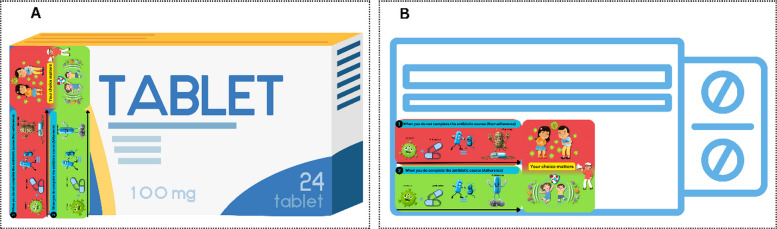
Application intervention sticker to antibiotic box **A** vertically and **B** horizontally

The intervention will be delivered as a sticker affixed to antibiotic packaging (boxes or packs) for patients in the intervention group, ensuring easy distribution during medication dispensing, as shown in Fig. 7. The sticker measures 2.5 × 6 cm, allowing for clear visibility and fitting on the box without obscuring important information. The patient will be monitored for medication dispensing at the hospital or nearby pharmacies and drug stores. This format ensures that the patient can take the information home, refer to it later, and be reminded of the importance of adherence after the consultation.

### Outcomes

#### Primary outcome

The primary outcome is antibiotic adherence, measured by tracking the pill count at the end of the prescribed antibiotic treatment. This will be expressed as a proportion (number of pills taken/total prescribed) and as a categorical variable (adhered vs. not adhered). Adherence is defined as taking ≥ 90% of the prescribed antibiotic course [32]. This will be assessed during a follow-up visit to the hospital or a home visit.

#### Secondary outcomes

##### UTI recurrence

This will be measured using a dichotomous question (yes, no). The physician will confirm the recurrence of UTI.

##### Knowledge and attitude

Knowledge and attitude are critical behavioral determinants of antibiotic adherence and clinical outcomes [33, 34]. Theoretical models such as the Knowledge, Attitudes, and Practice (KAP) model and the Theory of Planned Behavior (TPB) suggest that improved knowledge and positive attitudes toward antibiotic use influence adherence behaviors, which in turn reduce clinical risks such as UTI recurrence [33, 35].

Patient knowledge of antibiotic use, antibiotic resistance, and the importance of completing the treatment will be assessed using a 10-item questionnaire. The item was developed based on the previously reported studies [36–38]. The response will be collected as “true” and “false.” The true response will be scored 1 point and the incorrect response as 0. Attitude toward antibiotic use and adherence will be measured using a 5-item Likert scale, developed based on the previous studies [38–40], with responses ranging from 1 (strongly disagree) to 5 (strongly agree).

The content validity of the tools was established through expert review by two academicians having similar academic backgrounds, one pharmacist and one infectious disease specialist. Discrepancies in item wording were resolved by consensus. A pilot study was conducted with 60 patients. Preliminary reliability demonstrated acceptable internal consistency: Cronbach’s alpha for knowledge = 0.71, and for attitude = 0.73, showing valid internal consistency. Content Validity Index (CVI) analysis showed a scale-level CVI of 0.75 for knowledge and 0.83 for attitude, indicating acceptable validity.

### Data collection

#### Data collection procedure

Data will be collected utilizing EpiCollect5, a secure, cloud-based platform for mobile data collection that facilitates real-time monitoring and centralized management [41]. The principal investigator will oversee the entire data collection process. Three designated data collection supervisors will be appointed as “project managers” within the EpiCollect5 system. Each supervisor will be responsible for managing a team of trained data collectors operating at various field sites. The supervisors will monitor data quality, provide on-site guidance, and ensure compliance with standardized data collection protocols. The principal investigator will perform regular remote inspections via the EpiCollect5 dashboard to verify data completeness, consistency, and accuracy. This tiered supervision model enables prompt identification and resolution of field-level challenges, fosters data integrity, and guarantees the uniform application of procedures across sites. Each participant will be assigned a unique study code. Access to the project database is restricted to authorized study investigators using role-based permissions. All data collectors will undergo standardized training prior to field deployment to uphold inter-rater reliability and ethical standards.

#### Baseline data

At the start of the study, baseline demographic and patient data will be collected. Additionally, questionnaires regarding knowledge and attitudes will also be collected from all participants. During this stage, patients will be screened based on their prescription filling. Those who filled the prescription will be included for follow-up, while those who did not fill the prescription will be excluded, and the reasons for not filling the prescription will be identified. Data collectors will be trained for intervention implementation and data collection.

#### Follow-up data

**Fig. 8:**
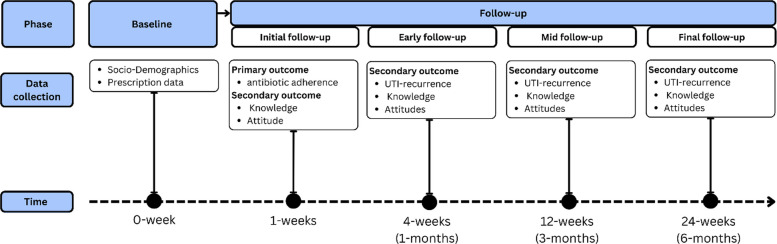
Time frame for data collection

**Fig. 9:**
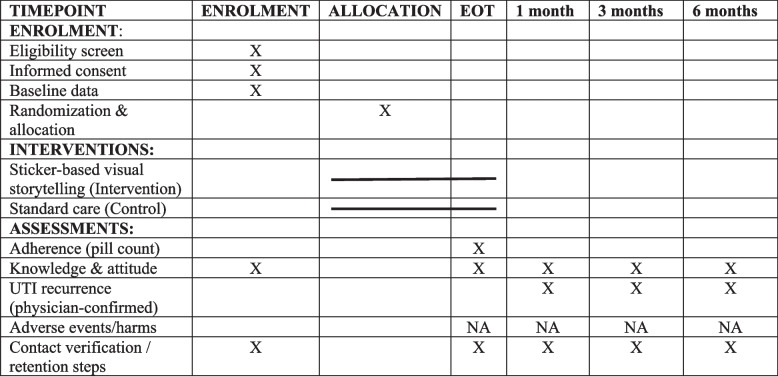
SPIRIT schedule of enrollment, interventions, and assessments. Adapted from SPIRIT 2025 guidelines [25]. Notes: X = assessment occurs at this timepoint; — = intervention delivery/exposure period. EOT = end of antibiotic treatment (typically day 3–7 depending on course). Follow-up windows: 1 month (± 7 days), 3 months (± 14 days), 6 months (± 21 days). NA not applicable (no biological specimens collected; harms not systematically assessed due to minimal-risk behavioral intervention)

At the follow-up stage, pill counts will measure adherence, and structured questionnaires will be administered to assess changes in knowledge and attitudes regarding antibiotic use. Then, the next follow-up will be done after 1 month, 3 months, and 6 months. At each follow-up, either at the hospital or home visit, the pill count will be performed by the trained data collectors. Returned blister packs will be checked against prescription records. Any discrepancies between the baseline dispensed quantity and the physical count will be documented in site monitoring logs and reviewed by the site supervisor. If packaging is not returned, adherence via pill count will be coded “not verifiable” and treated as missing for the primary pill-count outcome. The details are given in Fig. 8. The overall schedule of enrollment, interventions, and assessments is illustrated in Fig. 9.

### Statistical analysis plan

#### Primary outcome analysis

The primary outcome will be analyzed as a categorical variable (adhered vs. not adhered). The chi-square test or Fisher’s exact test (if expected counts are < 5) will be used for between-group differences. A mixed-effects logistic regression model will estimate the odds ratio (OR) for adherence in the intervention vs. control group, adjusted for baseline characteristics (e.g., age, sex, MPI category, education). Hospital will be included as a random intercept to account for clustering due to stratified block randomization by site.

Based on the multidimensional poverty index [5], which categorizes poverty into four subgroups, namely (1) not deprived, (2) vulnerable to deprivation, (3) deprived, and (4) severely deprived, a subgroup analysis will be carried out to assess the effect of intervention across these subgroups. Results will be reported as adjusted odds ratios (aORs) with 95% confidence intervals (CIs) and *p* values.

#### Secondary outcomes analysis

The knowledge and attitude scores will be summed for each participant. Descriptive statistics (mean, standard deviation) will be calculated for both groups, and the mean score will be compared using either independent samples *t*-tests (for normal distribution) or Mann-Whitney *U* tests (for non-normal distribution) for between-group comparison.

UTI recurrence will be a binary variable (yes/no). The chi-square test or Fisher’s exact test (if expected counts are < 5) will be used to compare the proportion of UTI recurrence between the intervention and control groups. Recurrence (yes/no) will be compared between groups using a mixed-effects logistic regression with hospital as a random intercept, adjusted for baseline characteristics. Results will be reported as aORs with 95% CIs.

When multiple comparisons are performed, the Bonferroni correction will be applied to control for the family-wise error rate, ensuring the results remain statistically robust.

#### Intention-to-treat (ITT) and per-protocol (PP) analysis

##### ITT analysis

The primary analysis will follow the intention-to-treat (ITT) principle and include all randomized participants. We will handle participants with lost to follow-up, missing outcomes, and covariate data using multiple imputations by chained equations (MICE) under a missing-at-random (MAR) assumption [42, 43]. The imputation model will include the primary outcome, baseline covariates predictive of outcome and missingness (age, sex, MPI category, baseline knowledge, and attitude scores). We will generate at least m = max (20, % missing × 1) imputations, fit the planned analysis model within each imputed dataset, and combine estimates using Rubin’s rules [44]. The participants who formally withdraw consent will be excluded from the analysis.

To assess robustness to departures from MAR, we will conduct sensitivity analyses: (1) a complete-case analysis; (2) pattern-mixture/delta-adjustment sensitivity analyses that impose plausible shifts (delta) to imputed outcomes under missing-not-at-random (MNAR) scenarios; and (3) a conservative worst-case/best-case bounds analysis for the primary binary outcome. Results from sensitivity analyses will be reported alongside the primary multiple imputation results and used to assess the robustness of conclusions regarding treatment effect.

##### Per-protocol analysis

In addition to the ITT analysis, a PP analysis will be conducted to assess the intervention’s effects on those who adhered to the protocol. This analysis will provide insight into the maximum impact of the intervention when implemented without deviations.

### Ethical considerations

#### Informed consent

Informed consent will be obtained from each participant by a trained data collector at each hospital site, ensuring that they receive a clear explanation of the study’s purpose (improving antibiotic adherence through VISTA intervention), procedures (randomization, intervention delivery, follow-up visits for pill counts, and questionnaire survey), and potential risks and benefits. Participants will be explicitly assured that their participation is entirely voluntary, and they retain the right to withdraw from the study at any time without penalty or impact on their medical care. Additionally, the consent process will describe data anonymization measures, such as replacing personal identifiers with unique codes, and confidentiality safeguards, including secure storage of data and restricted access to authorized study personnel only.

#### Ethical approval

The Ethics Committee of the Department of Pharmacy Practice, Bahauddin Zakariya University, Multan (Approval No. BZU-FOPDPP-2466) and Saidu Group of Teaching Hospitals, Swat (Approval No. 1401/03) reviewed and approved the study.

Any important protocol amendments, such as changes to eligibility criteria, outcomes, or analyses, will be reviewed and approved by the relevant ethics committees, updated in ClinicalTrials.gov, and communicated to all investigators and participating sites.

#### Study registration

The trial was registered with ClinicalTrials.gov on March 3, 2025, with trial ID: NCT06885658 (https://register.clinicaltrials.gov/prs/beta/records).

### Qualitative analysis

A qualitative study will examine the perceived mechanisms through which the VISTA intervention influences antibiotic adherence, focusing on participants’ experiences, behavioral pathways, and contextual factors.

A qualitative descriptive approach will be employed. Semi-structured interviews will be conducted with purposefully selected patients. Sample size will be determined based on thematic saturation. Participants will be selected from the intervention group for post-intervention delivery and adherence assessment. Patients who completed the follow-up for adherence measurement and expressed willingness to participate in the study will be included. Semi-structured interviews will follow an interview guide based on the Theoretical Domains Framework (TDF) [33]. TDF will assist in identifying the behavioral barriers and enablers affecting antibiotic adherence. This allows the interview guide to explore the multifaceted factors influencing adherence. The interviews will be conducted in the local language (Urdu/Pashto), audio-recorded, and transcribed into English.

The qualitative data will be analyzed using thematic analysis. The transcripts will be coded using TDF domains to identify patterns. Emergent themes will be integrated with TDF themes. This sub-study will provide actionable insights into how and why the intervention works, ensuring its theoretical coherence and contextual relevance. Findings will guide refinements for scalability in low-resource settings and inform future implementation strategies.

## Discussion

This study aims to investigate the efficacy of visual storytelling as an intervention to enhance antibiotic adherence among patients diagnosed with UTIs. The potential of visual storytelling to impact health behavior is based on its capability to bridge the gaps between knowledge and action.

Storytelling can enhance engagement and promote health behavior change. Nudge theory explains that the way choices are presented significantly nudges behavior and can lead to desired behavioral actions [45]. Narrative theory explains that stories can influence attitudes by encouraging emotional transportation, where individuals become mentally absorbed in a narrative, resulting in changes in belief and behavior [15]. Visual storytelling adds an extra layer, amplifying this effect by incorporating visual elements that appeal to both cognitive and emotional processing, thereby improving the retention and recall of health messages and behaviors. In the context of antibiotic adherence, visual storytelling aims to address cognitive and emotional barriers that typically hinder adherence, such as lack of understanding, forgetfulness, and resistance to taking medication as prescribed. By presenting the outcomes of choices offered for antibiotic adherence, this intervention will encourage patients to recognize the importance of completing their antibiotic course, thereby reducing the risk of antibiotic resistance. VISTA intervention aligns well with goals to improve antibiotic adherence in UTI patients through patient-centered engagement. For instance, a recent review showed that storytelling improved self-management and peer support in chronic disease patients by fostering self-efficacy [16]. Similarly, digital storytelling has been shown to improve emotional engagement and understanding among immigrants and the refugee population, which can motivate patients to adhere to treatment plans [46]. Furthermore, combining multimedia in visual storytelling improves comprehension of complex health information [47], supporting the use of this approach to emphasize the importance of completing antibiotic courses and mitigating non-compliance. However, in Pakistan, in contexts like Pakistan, where nearly half of the population lacks mobile access [23], the feasibility of digital storytelling intervention may be limited. To overcome this barrier while preserving the benefits of storytelling, we have developed a sticker-based VISTA intervention to improve patients’ adherence to antibiotics.

The primary outcome of the trial will be adherence to antibiotic regimens among UTI patients, as measured by pill counts. Secondary outcomes will include improvements in patient knowledge regarding antibiotic use, changes in attitudes toward antibiotic resistance, and increased patient confidence in managing their health. As demonstrated in other health-related storytelling interventions, visual stories that emphasize the emotional and practical aspects of behavior change, such as the potential consequences of not completing antibiotic courses, are more likely to lead to sustained changes in behavior [48]. The intervention will also aim to increase patient self-efficacy, which is often a predictor of health behavior change. By providing patients with the outcomes of their choices, we anticipate an increase in their ability to adhere to prescribed regimens.

The use of VISTA intervention to improve antibiotic adherence is a novel approach, bridging behavioral science and clinical practice. While storytelling has proven effective in other health domains, such as vaccination promotion and chronic disease management [16, 47, 48], its application to antibiotic adherence is underexplored. This tool could be easily adopted by policymakers into national antimicrobial stewardship programs, while clinicians could incorporate it into patient counseling. Empirical validation of its efficacy within the UTI cohort will demonstrate its translational potential, enabling adaptation to other prevalent infections, such as respiratory tract infections. By facilitating a transformative approach to antimicrobial stewardship, this intervention aligns with global priorities for combating antibiotic resistance while addressing critical gaps in low-resource healthcare systems.

This study presents numerous methodological and practical strengths. Foremost, its RCT design guarantees a rigorous assessment of the intervention’s efficacy, thereby minimizing bias through the processes of randomization and the blinding of outcome assessors. The incorporation of nudge theory provides robust theoretical support, which enhances the intervention’s capacity to foster significant behavior modification. Moreover, the development of the intervention entailed a structured two-step methodology, which included expert consensus via the Delphi technique and soliciting patient feedback. This comprehensive approach ensures cultural relevance, clarity, and practicality for implementation in real-world settings. The employment of objective adherence measures, such as pill counts, alongside blind physician evaluations of UTI recurrence, further fortifies the validity of the findings. Lastly, the intervention’s cost-effective, sticker-based format renders it scalable and adaptable to a range of healthcare environments, aligning with the WHO’s priorities regarding antimicrobial stewardship in resource-constrained regions [49].

While this study addresses critical gaps in antibiotic adherence research, certain limitations merit consideration. First, pill counts, though objective, may not fully capture intentional non-adherence; for example, skipped doses despite retained pills. However, the analysis of the UTI recurrence can indirectly assess the effectiveness of the intervention. Second, the regional focus on tertiary hospitals in Pakistan may limit generalizability to other populations. However, the inclusion of culturally tailored content, refined through Delphi consensus and patient feedback, enhances relevance to similar low-resource settings. Third, external factors such as medication access or healthcare provider communication could influence adherence. While these variables are challenging to control in real-world trials, the feasibility study explicitly evaluates logistical barriers (e.g., recruitment rates, sticker delivery fidelity) to optimize implementation. Fourth, the 6-month follow-up period may not fully capture long-term adherence patterns or ABR outcomes. Finally, there is potential social desirability bias in self-reported knowledge and attitude responses.

Implementation challenges may arise related to the development, printing, and distribution logistics of intervention materials, variability in staff training and engagement, and maintaining intervention fidelity across diverse study sites. Ensuring consistent supervision, particularly in resource-limited hospital settings, will be essential. Addressing these operational factors early will be crucial for the sustainability, scalability, and policy translation of the VISTA intervention.

If effective, the VISTA intervention can be integrated into provincial and AMR programs through collaboration with the Ministry of National Health Services, Regulations and Coordination (MoNHSRC) and provincial health departments. Integration into community pharmacy and primary-care education platforms, supported by existing AMR stewardship initiatives, would allow wider adoption.

## Dissemination

The primary results will be disseminated through a peer-reviewed journal focused on antimicrobial resistance to support an antimicrobial stewardship policy and practice. To translate evidence into practice, a policy brief will be developed and submitted to the Ministry of Health Service, Regulation and Coordination, Pakistan, emphasizing the scalability of the nudge-based VISTA intervention in low-resource settings.

## Trial status

Protocol version 2, approved March 11, 2025. Recruitment started on August 06, 2025, and the anticipated recruitment completion date will be September 30, 2025.

## Supplementary Information

Additional file 1: SPIRIT checklist.

## Data Availability

Not applicable.
