# Supplementary material for: Effectiveness of nudge-based visual storytelling in antibiotic adherence in uncomplicated urinary tract infection in Pakistan: protocol for a randomized controlled trial
Source: Trials. 2025 Dec 17;27:63. doi: 10.1186/s13063-025-09328-1 (PMC12822019; doi:10.1186/s13063-025-09328-1)
Supplement: Supplementary file 1 — Additional file 1: SPIRIT checklist. [file 13063_2025_9328_MOESM1_ESM.docx]

**SPIRIT 2025 checklist of items to address in a randomized trial protocol**

| **Section / Topic** | **No** | **SPIRIT 2025 checklist item description** | **Reported on page no.** |
| --- | --- | --- | --- |
| **Administrative information** | | |  |
| Title and structured summary | 1a | Title stating the trial design, population, and interventions, with identification as a protocol | Title page – p. 1 |
|  | 1b | Structured summary of trial design and methods, including items from the World Health Organization Trial Registration Data Set | Abstract – p. 3 – 4 |
| Protocol version | 2 | Version date and identifier | Trial status – p. 23 |
| Roles and responsibilities | 3a | Names, affiliations, and roles of protocol contributors | Title page – p. 1, Author contribution – p, 27 |
|  | 3b | Name and contact information for the trial sponsor | Trial sponsor – p. 26 |
|  | 3c | Role of trial sponsor and funders in design, conduct, analysis, and reporting of trial; including any authority over these activities | Funding– p. 26 |
|  | 3d | Composition, roles, and responsibilities of the coordinating site, steering committee, endpoint adjudication committee, data management team, and other individuals or groups overseeing the trial, if applicable | N/A – No independent committees; oversight by PI and site supervisors |
| **Open science** | | |  |
| Trial registration | 4 | Name of trial registry, identifying number (with URL), and date of registration. If not yet registered, name of intended registry | Trial registration – p. 4 |
| Protocol and statistical analysis plan | 5 | Where the trial protocol and statistical analysis plan can be accessed | The statistical analysis plan is described in the Methods section (p. 16 – 18)). The full protocol can be accessed through the ClinicalTrials.gov registration (NCT06885658), as provided in the trial information (p. 4). |
| Data sharing | 6 | Where and how the individual de-identified participant data (including data dictionary), statistical code, and any other materials will be accessible | Individual de-identified participant data will not be publicly available due to ethical restrictions. Only aggregate results will be reported in publications and trial registries. The statistical analysis plan is provided in the Methods section (p. 16- 18). |
| Funding and conflicts of interest | 7a | Sources of funding and other support (e.g., supply of drugs) | Funding– p. 26 |
|  | 7b | Financial and other conflicts of interest for principal investigators and steering committee members | No competing interests declared – p. 26 |
| Dissemination policy | 8 | Plans to communicate trial results to participants, healthcare professionals, the public, and other relevant groups (e.g., reporting in trial registry, plain language summary, publication) | Plans for peer-reviewed publication, policy brief to Ministry of Health – p. 25 |
| **Introduction** | | |  |
| Background and rationale | 9a | Scientific background and rationale, including summary of relevant studies (published and unpublished) examining benefits and harms for each intervention | Background– p. 5–7 |
|  | 9b | Explanation for choice of comparator | Study design – page 8 |
| Objectives | 10 | Specific objectives related to benefits and harms | Research question/aims framed using FINER criteria – p. 7 |
| **Methods: Patient and public involvement, trial design** | | |  |
| Patient and public involvement | 11 | Details of, or plans for, patient or public involvement in the design, conduct, and reporting of the trial | Patients involved in the intervention development via understanding study; Delphi panel included experts – p. 9-12 |
| Trial design | 12 | Description of trial design including type of trial (e.g., parallel group, crossover), allocation ratio, and framework (e.g., superiority, equivalence, non-inferiority, exploratory) | Parallel, two-arm RCT, 1:1 allocation, superiority – p. 7 – 8 |
| **Methods: Participants, interventions, and outcomes** | | |  |
| Trial setting | 13 | Settings (e.g., community, hospital) and locations (e.g., countries, sites) where the trial will be conducted | Six tertiary hospitals in Khyber Pakhtunkhwa – p. 8 |
| Eligibility criteria | 14a | Eligibility criteria for participants | Eligibility: adults ≥18 with uncomplicated UTI; exclusions listed – p. 8 – 9 |
|  | 14b | If applicable, eligibility criteria for sites and for individuals who will deliver the interventions (e.g., surgeons, physiotherapists) | N/A – No additional site/provider eligibility beyond willingness and diagnosis |
| Intervention and comparator | 15a | Intervention and comparator with sufficient details to allow replication including how, when, and by whom they will be administered. If relevant, where additional materials describing the intervention and comparator (e.g., intervention manual) can be accessed | Sticker-based visual storytelling vs standard care – p. 9 – 12 |
|  | 15b | Criteria for discontinuing or modifying allocated intervention/comparator for a trial participant (e.g., drug dose change in response to harms, participant request, or improving/worsening disease) | Patient population and sample size – p. 9 |
|  | 15c | Strategies to improve adherence to intervention/comparator protocols, if applicable, and any procedures for monitoring adherence (e.g., drug tablet return, sessions attended) | SEAR framework, pill counts– p. 9, 14 |
|  | 15d | Concomitant care that is permitted or prohibited during the trial | N/A – No restrictions on concomitant care |
| Outcomes | 16 | Primary and secondary outcomes, including the specific measurement variable (e.g., systolic blood pressure), analysis metric (e.g., change from baseline, final value, time to event), method of aggregation (e.g., median, proportion), and time point for each outcome | Outcomes: adherence (primary); recurrence, knowledge, attitudes (secondary) – p. 14 – 15 |
| Harms | 17 | How harms are defined and will be assessed (e.g., systematically, non-systematically) | N/A – No anticipated harms; behavioral intervention only |
| Participant timeline | 18 | Time schedule of enrollment, interventions (including any run-ins and washouts), assessments, and visits for participants. A schematic diagram is highly recommended (see Figure) | Schedule: enrolment, allocation, follow-up at end of treatment, 1m, 3m, 6m – p. 16 (Fig 9) |
| Sample size | 19 | How sample size was determined, including all assumptions supporting the sample size calculation | Sample size 358; pilot effect size, 80% power, α=0.05, 15% attrition – p. 9 |
| Recruitment | 20 | Strategies for achieving adequate participant enrollment to reach target sample size | Recruitment via SEAR framework in urology OPDs – p. 9 |
| **Methods: Assignment of interventions** | | |  |
| Randomization: |  |  |  |
| Sequence generation | 21a | Who will generate the random allocation sequence and the method used | Random allocation sequence generated in R (blockrand) – p. 12 |
|  | 21b | Type of randomization (simple or restricted) and details of any factors for stratification. To reduce predictability of a random sequence, other details of any planned restriction (e.g., blocking) should be provided in a separate document that is unavailable to those who enroll participants or assign interventions | Stratified block randomization by hospital, block size 6 – p. 12 |
| Allocation concealment  mechanism | 22 | Mechanism used to implement the random allocation sequence (e.g., central computer/telephone; sequentially numbered, opaque, sealed containers), describing any steps to conceal the sequence until interventions are assigned | Allocation concealment with sequential number – p. 13 |
| Implementation | 23 | Whether the personnel who will enroll and those who will assign participants to the interventions will have access to the random allocation sequence | data collection – p. 15 – 16 |
| Blinding | 24a | Who will be blinded after assignment to interventions (e.g., participants, care providers, outcome assessors, data analysts) | Outcome assessors/analysts blinded; participants/staff unblinded – p. 13 |
|  | 24b | If blinded, how blinding will be achieved and description of the similarity of interventions | Data coded Group A/B; physicians blinded – p. 13 |
|  | 24c | If blinded, circumstances under which unblinding is permissible, and procedure for revealing a participant’s allocated intervention during the trial | N/A – Behavioral trial; unblinding not required |
| **Methods: Data collection, management, and analysis** | | |  |
| Data collection methods | 25a | Plans for assessment and collection of trial data, including any related processes to promote data quality (e.g., duplicate measurements, training of assessors) and a description of trial instruments (e.g., questionnaires, laboratory tests) along with their reliability and validity, if known. Reference to where data collection forms can be accessed, if not in the protocol | Data collection - p. 16 -17 |
|  | 25b | Plans to promote participant retention and complete follow-up, including list of any outcome data to be collected for participants who discontinue or deviate from intervention protocols | Follow-up assessments are scheduled at end of treatment, 1, 3, and 6 months (pill count, knowledge, attitudes, UTI recurrence) – p. 16 |
| Data management | 26 | Plans for data entry, coding, security, and storage, including any related processes to promote data quality (e.g., double data entry; range checks for data values). Reference to where details of data management procedures can be accessed, if not in the protocol | Data managed in EpiCollect5; weekly monitoring by supervisors – p. 15 – 16 |
| Statistical methods | 27a | Statistical methods used to compare groups for primary and secondary outcomes, including harms | Statistical methods: Chi-square/Fisher, mixed-effects regression – p. 16 - 18 |
|  | 27b | Definition of who will be included in each analysis (e.g., all randomized participants), and in which group | Analyses: ITT and PP – p. 18 |
|  | 27c | How missing data will be handled in the analysis | Missing data handled with multiple imputation (MICE) – p. 18 |
|  | 27d | Methods for any additional analyses (e.g., subgroup and sensitivity analyses) | Additional analyses: subgroup by poverty, sensitivity analyses – p. 17 |
| **Methods: Monitoring** | | |  |
| Data monitoring committee | 28a | Composition of data monitoring committee (DMC); summary of its role and reporting structure; statement of whether it is independent from the sponsor and funder; conflicts of interest and reference to where further details about its charter can be found, if not in the protocol. Alternatively, an explanation of why a DMC is not needed | N/A – No DMC; minimal-risk trial, oversight by PI |
|  | 28b | Explanation of any interim analyses and stopping guidelines, including who will have access to these interim results and make the final decision to terminate the trial | N/A – No interim analyses or stopping guidelines |
| Trial monitoring | 29 | Frequency and procedures for monitoring trial conduct. If there is no monitoring, give explanation | Monitoring weekly by supervisors; PI oversight – p. 15 – 16 |
| **Ethics** | | |  |
| Research ethics approval | 30 | Plans for seeking research ethics committee/institutional review board approval | Ethics approvals from BZU and Saidu Hospital committees – p. 19 |
| Protocol amendments | 31 | Plans for communicating important protocol modifications to relevant parties | Ethical consideration – p. 21 |
| Consent or assent | 32a | Who will obtain informed consent or assent from potential trial participants or authorized proxies, and how | Ethical consideration – p. 20 |
|  | 32b | Additional consent provisions for collection and use of participant data and biological specimens in ancillary studies, if applicable | N/A – No biological specimens collected |
| Confidentiality | 33 | How personal information about potential and enrolled participants will be collected, shared, and maintained in order to protect confidentiality before, during, and after the trial | Confidentiality: anonymized, coded, secure storage – p. 19 |
| Ancillary and post-trial care | 34 | Provisions, if any, for ancillary and post-trial care, and for compensation to those who suffer harm from trial participation | N/A – No ancillary/post-trial care required; behavioral trial |
